# Traumatic Pulmonary Pseudocyst Mimicking a Congenital Cystic Lung Disease

**DOI:** 10.1155/2018/7269694

**Published:** 2018-07-11

**Authors:** Amjad Kanj, Hussam Tabaja, Ayman O. Soubani, Nadim Kanj

**Affiliations:** ^1^Wayne State University, Department of Internal Medicine, USA; ^2^Wayne State University, Division of Pulmonary Critical Care and Sleep Medicine, USA; ^3^American University of Beirut, Division of Pulmonary and Critical Care, Lebanon

## Abstract

Traumatic pulmonary pseudocyst (TPP) is a rare entity that occurs following a trauma to the chest. It usually presents as multiple cystic lesions on thoracic imaging. It is treated conservatively and tends to completely resolve after few months. In the absence of striking signs of trauma such as rib fractures, TPP can be mistaken for other cystic lung diseases. We present a case of TPP in a 17-year-old male who was seen for mild hemoptysis after falling off a cliff. The extent of his right lower lobe cystic lesions along with the lack of major signs of trauma led to an incorrect diagnosis of congenital pulmonary airway malformation. The patient was considered for lobectomy, which he refused. Imaging of the chest repeated one and three years later showed complete resolution of the lesions.

## 1. Introduction

Traumatic pulmonary pseudocysts (TPPs) are cystic and cavitary lesions that can develop in the lung parenchyma after a trauma to the chest [1]. Although TPP remains a rare entity, awareness of this benign condition is imperative as it may mimic other more serious pulmonary cystic diseases. [2] We present a case where TPP was initially misdiagnosed as congenital pulmonary airway malformation (CPAM) and highlight the potential consequence of such misdiagnosis.

## 2. Case Report

**Figure 1:**
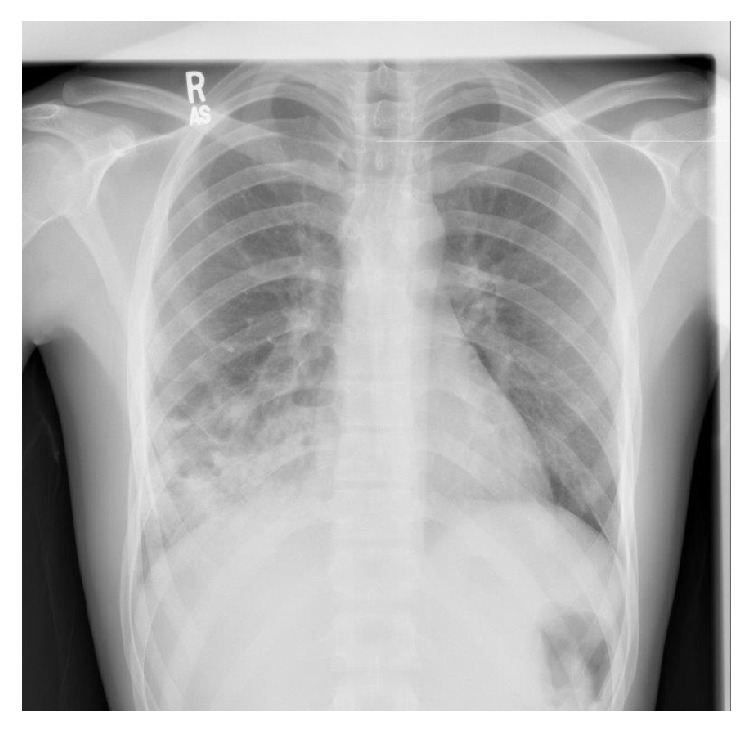
Chest X-ray obtained 2 hours after the trauma showing multiple cystic lesions containing air-fluid levels.

**Figure 2:**
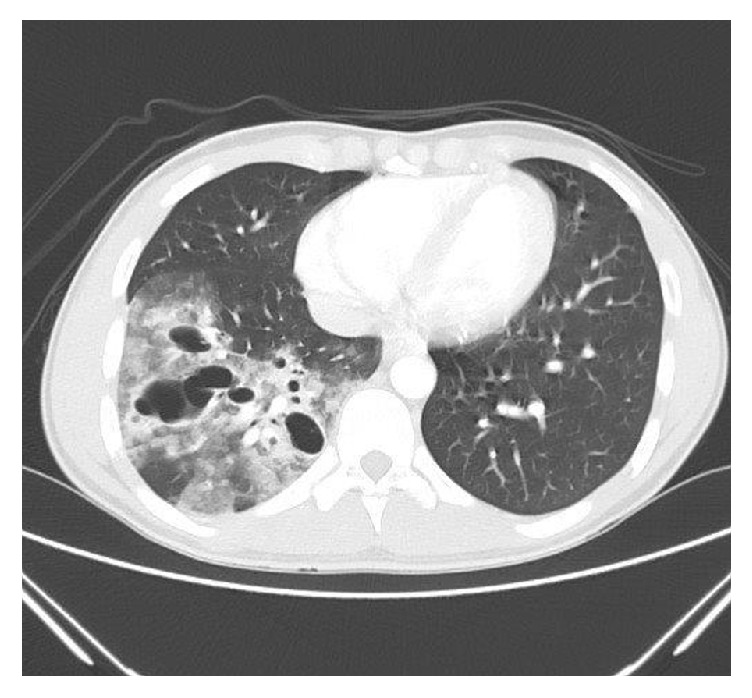
CT scan of the chest showing thick-walled multicystic lesions with patchy air space opacities.

A previously healthy 17-year-old male presented with the complaint of mild hemoptysis after sustaining a blunt trauma to the chest. He fell off a 3-foot cliff while hiking and landed on the right side of his chest. On presentation, the patient's pain was tolerable and he was breathing comfortably. His vital signs showed a pulse of 98 beats per minute, blood pressure of 110/60 mmHg, and an oxygen saturation of 98% on room air. His exam revealed minimal abrasions, ecchymosis, and tenderness over the right lower chest wall at the anterior axillary line. His lung exam revealed decreased breath sounds over the right lower lung field. A chest X-ray obtained within 2 hours of the trauma showed alveolar opacities in the right lower lobe with multiple cystic air spaces containing air-fluid levels (**Figure 1**). There were no associated pleural effusions, pneumothorax, or rib fractures. A Computed Tomography (CT) scan of the chest showed thick-walled multicystic lesions with patchy air space opacities and consolidations in the right lower lobe (**Figure 2**). No previous chest imaging was available for comparison. The described CT scan abnormalities, in the absence of extrapulmonary posttraumatic findings, were suggestive of CPAM with superimposed bleeding. The patient was admitted for observation and evaluation and placed on intravenous Amoxicillin/Clavulanate. Spirometry done the next day was normal. His complete blood count, basic metabolic panel and bleeding profile were normal. His C-reactive protein was elevated at 32.0 mg/L. Gram stain, acid fast stain, and sputum cultures for bacteria, fungi, and tuberculosis were all negative. Alpha-1 antitrypsin and immunoglobulin levels were within normal limits.

**Figure 3:**
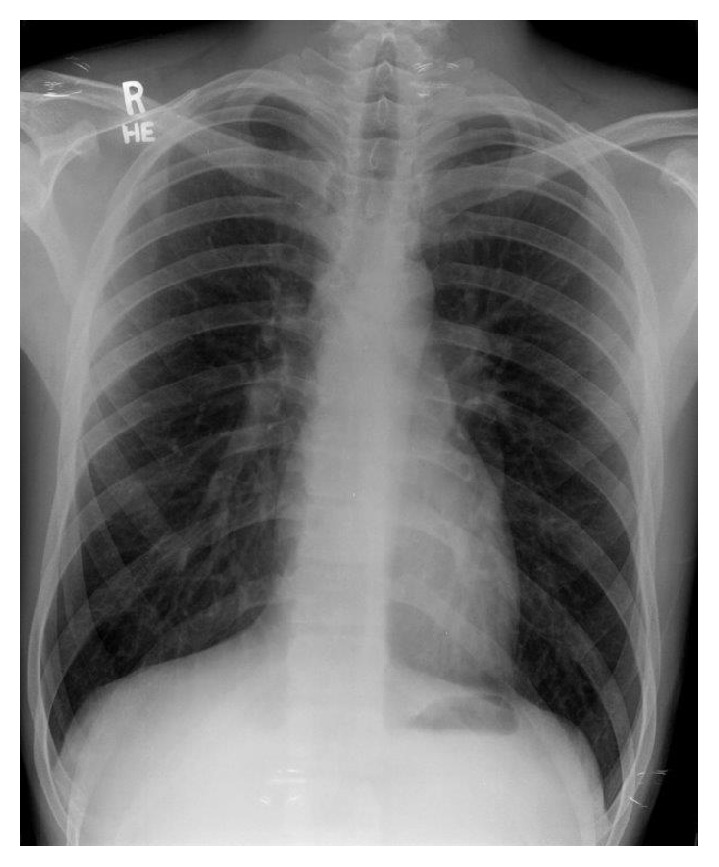
Chest X-ray of patient repeated 1 year later showing complete resolution of the cystic lesions.

**Figure 4:**
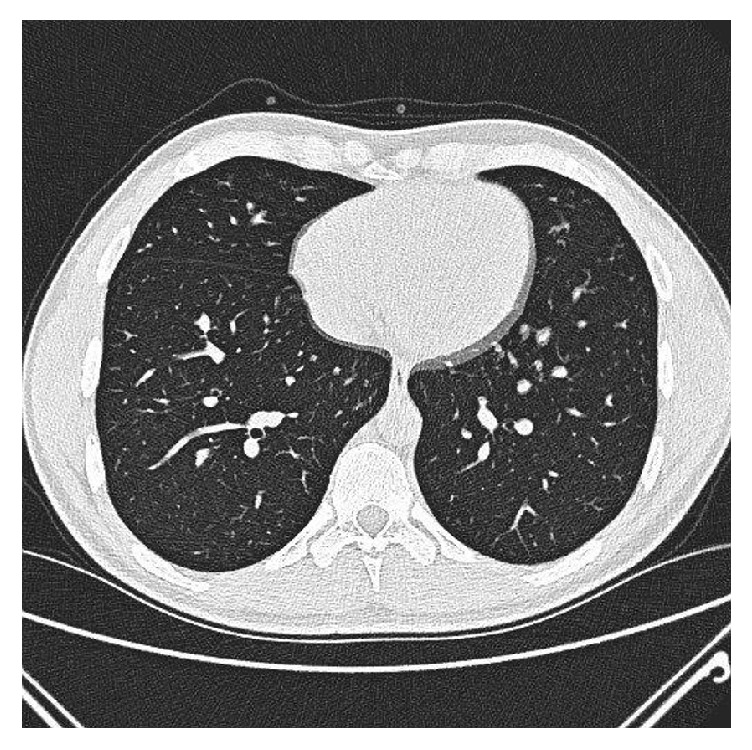
A normal chest CT scan of the patient obtained three years after the trauma.

The patient was evaluated by a cardiothoracic surgeon and a right lower lobectomy was being considered. However, given the indolent course of his disease and his negative history for pulmonary infections thus far, the patient elected to defer further surgical evaluation and, instead, follow-up with clinical observation. He remained asymptomatic throughout the interval period and a chest X-ray repeated after one year was normal (**Figure 3**). Finally, a CT scan of the chest obtained two years later showed complete resolution of the previously described abnormalities (**Figure 4**). Due to the fact that his cysts resolved spontaneously with time after his trauma, the patient was finally diagnosed with TPP.

## 3. Discussion

TPP develops as a result of high compressive forces transmitted to the pulmonary parenchyma during chest wall trauma. It is primarily described following a blunt trauma to the chest but is also seen with penetrating injuries [3]. The rapid compression and decompression of the chest result in lacerations and cavitary lesions within the lung tissue, particularly when the lungs are compressed against a closed glottis impeding the rapid ejection of air through the upper airways. Subsequently, if no communication exists between the cavities and the pleural space, air and fluid escape from the parenchyma and fill up the cavity [2].

Clinically, TPP can vary widely in presentation. Patients can be entirely asymptomatic or can have life-threatening respiratory compromise. Commonly reported symptoms include hemoptysis, which is seen in almost half of the patients. Other manifestations include shortness of breath, cough, chest pain, fevers, and leukocytosis. Additionally, TPP is almost always associated with rib fractures or other evidences of trauma such as large contusions and pneumothoraces [4].

Radiologically, cavitary lesions in TPP appear on chest X-ray within the first 24 hours of trauma in almost half the patients. The size of the pseudocysts ranged from 2 to 14 cm in diameter in previous reports. [2] They can be single or multiple and can be seen either on the same side of trauma or on the opposite side as a result of contrecoup injury [5]. Notably, TPP lesions can change quickly in size and shape on serial X-rays done over the course of days. This can help distinguish TPP from other cystic conditions. An even more sensitive imaging modality is CT scan of the chest. The finding of single or multiple cysts with thin walls along with air space consolidation of the surrounding parenchyma has been suggested as diagnostic in the setting of a preceding chest wall trauma [6].

TPP is usually self-remitting and the overall prognosis of the condition itself is excellent. Complications are rare but may include infection, pneumothorax, or bleeding [7]. The rate of complications increases when the patient is exposed to unnecessary procedures from failure to accurately diagnose this condition. Our patient, for instance, was being considered for what turned out to be an unnecessary surgical lobectomy. This highlights the importance of differentiating TPP from other resembling yet more serious conditions.

In our patient, the extent of the intraparenchymal lesions along with the absence of extrapulmonary posttraumatic findings led to the erroneous diagnosis of CPAM. Furthermore, antibiotics were started for concern of a superimposed infectious process, in the setting of active hemoptysis and right lower lobe opacities and consolidations on chest imaging. CPAM, formerly known as congenital cystic adenomatoid malformation (CCAM), is a unilateral congenital lung disease in which one or multiple cysts replace a lobe of the lungs. It is the most common developmental malformation of the lungs. Cavities are often thick-walled and can range in size from less than 1 cm to more than 2 cm depending on the type of lesions [8].

The pathophysiology of CPAM is unclear, but the condition is thought to arise from an airway obstruction during fetal development [9]. It generally leads to respiratory distress and infections in neonates and infants, and less than 10% of patients will present after the age of 1 year. In adults, it may manifest as recurrent or persistent pneumonia in the same location. Other features may include lung abscesses, failure to thrive, or a pneumothorax. The decision for elective surgery in asymptomatic patients with CPAM stems from its potential for complications as well as a possible association with lung malignancy [10].

CT scan remains the gold standard for diagnosing both CPAM and TPP. Unfortunately, there are no specific radiological findings that help distinguish both entities. Cystic lesions typically affect the lower lobes of the lungs in CPAM and TPP and can be single or multiple and unilateral or, rarely, bilateral. Furthermore, the cysts can be filled with air or fluids in both conditions. Nevertheless, TPP becomes more plausible in the presence of preceding trauma. Furthermore, regression of the lesions on repeat chest imaging is a key diagnostic finding that occurs only in TPP as opposed to CPAM. For this reason, patients suspected to have TPP ought to have repeat images demonstrating regression of the lung lesions before a definite diagnosis of TPP is made. The average time for cyst resolution in TPP is estimated at 4 months [11].

## 4. Conclusion

Unlike CPAM that often requires surgical resection, TPP is a benign, self-resolving cystic lung condition that develops after thoracic trauma. Physicians should be cognizant of TPP as an improper diagnosis can expose the patient to unnecessary drugs, procedures, or even surgery.
